# The temporal development of memory processes in source monitoring: An investigation with mouse tracking

**DOI:** 10.3758/s13423-023-02289-z

**Published:** 2023-05-03

**Authors:** Hilal Tanyas, Beatrice G. Kuhlmann

**Affiliations:** School of Social Sciences, Department of Psychology, University of Mannheim, Mannheim, Germany

**Keywords:** Source monitoring, Source memory, Item memory, Temporal development, Mouse-tracking

## Abstract

The present study investigated whether we first remember an item (e.g., a word itself) and then its source (e.g., position on the screen) or whether the retrieval of item and source information can (partially) overlap. Participants were tested on the source either in immediate sequence to item recognition (as standard in source-monitoring research) or following as a separate block after full completion of the item recognition test to separate these processes in time, providing a baseline. Using the mouse-tracking procedure during the item and source tests, we analyzed how item and source decisions unfolded qualitatively over time. Despite no significant difference in the aggregated trajectory curvatures, more thorough analyses based on the individual trajectories revealed differences across the test formats. In the standard format, trajectories were less curved in the source than in the item test. In contrast, in the blocked format, this difference was in the other direction with source showing more curved trajectories than item. Alternative interpretations of mouse-trajectory curvatures on the source-monitoring paradigm and what their difference may imply for item and source processing are discussed.

**Supplementary Information:**

The online version contains supplementary material available at 10.3758/s13423-023-02289-z.

## Introduction

*Source monitoring* encompasses memory and judgment processes by which memory records are attributed to their origins (Johnson et al., 1993). Thereby, *source* refers to episodic details that denote the contextual circumstances under which the information itself was acquired. Our focus herein is memory processing in source monitoring, which demands both recognizing the previously encountered items (item memory, e.g., *what* was seen?) and discriminating the origin of those encountered items (source memory, e.g., *where* was it seen?).

Item and source memory are dissociated on a behavioral and neuropsychological level (e.g., Lindsay & Johnson, 1991; Mitchell & Johnson, 2009). However, we do not know yet whether they are also dissociated in time. To date, Johnson et al. (1994) addressed the time-course of *reality monitoring* (a special case of source monitoring, i.e., differentiating internal sources (e.g., imagined events) from external sources (e.g., perceived events)), and found that item recognition was available at earlier response lags than source discrimination. Using a similar response-lag procedure, Spaniol and Bayen (2002) compared the time-courses of item memory and source guessing in the absence of source memory in an external source-monitoring paradigm. However, we are not aware of a study tracking the spontaneous time-courses of item and source memory for external sources. On a theoretical level, Lindsay (2008) speculated about two possible serial time-courses in source monitoring in which either source retrieval may start only after item retrieval finishes, or, alternatively, the source is retrieved first and then provides information for item memory. There is indeed much research and debate on the possible *serial* time-courses of item and source memory (e.g., Bell et al., 2017; Fox & Osth, 2022; Malejka & Bröder, 2016; Starns et al., 2008). Yet, we are not aware of any work querying the possible alternative of *parallel* processing of item and source memory.

The standard source-monitoring test formats either ask for the item and source decision in one step (i.e., Was this item studied in source A, source B, or is it new?) or the source is queried in immediate succession to an “old” response for an item (cf. Marsh et al., 2006). Unpublished response-time data from our lab (Tanyas et al., 2022) frequently shows very fast responses on a source query immediately following an “old” judgment, suggesting that participants already retrieved the source during the preceding item query. That is, retrieval of item and source memory may not necessarily occur in a fully sequenced way, despite being probed in that order by the standard testing. Instead, source retrieval may already begin during item retrieval within the same test stage, indicating some degree of “partial overlap.”

## Mouse-tracking of memory processes

Mouse-tracking is a means to capture continuous neuronal activity in behavior (Spivey & Dale, 2006), and it has become a prominent analytic technique to gain insight into cognition (Freeman, 2018). In this procedure, participants decide between two spatially separated response options on the screen. Meanwhile, their mouse movements are continuously recorded. Tracking cursor positions makes it possible to measure response dynamics in different facets (for an overview of mouse-tracking metrics, see Kieslich et al., 2019).

In recent years, mouse tracking has also been employed in some studies investigating memory via recognition tasks requiring mouse responses. The multifaceted measures of mouse tracking allow researchers to test predictions from different aspects altogether (Gatti et al., 2022) or enable a breakdown of processes subserving recognition. For example, certain metrics of the mouse trajectories can be linked to response bias or encoding strength (Koop & Criss, 2016), while other metrics are related to metacognitive confidence (Papesh & Goldinger, 2012) and inherent memorability (Papesh et al., 2019). Critical to our interest, the pioneering work of L. Wulff and Scharf (2020) implemented mouse tracking to source monitoring and showed that trajectory curvature measured with the MAD (i.e., maximum absolute deviation toward the non-chosen option; Kieslich et al., 2019) is linked to *source memory*. Further, trajectory curvature measured with the maximum deviation from the direct path was also previously assessed in old/new judgments (cf. Gatti et al., 2022). In the following, we thus focus on the MAD considering previous applications of curvature metrics to old/new judgments and, more importantly, its link to source memory.

## Overview of the current study

To what extent should memory be detailed to differentiate between alternating response options? As conceptualized by Johnson et al. (1993), it differs by memory tasks, such that source monitoring needs even more differentiation than old-new recognition. Further, they suggest that differentiation of (item and source) memory dynamically changes and develops over time. Here, mouse tracking is a crucial technique to measure such *dynamic* processes, rather than showing only the end-product, by capturing how straighforwardly one opts for a certain response. Thus, we investigated temporal dynamics of item and source memory with mouse movements and specifically assessed trajectory curvature measured with the MAD.

To our knowledge, we are the first study to track item versus source *memory* courses in a standard external source-monitoring paradigm and the first to do so by applying mouse tracking. We manipulated different source-monitoring test formats (the *standard* sequential and *blocked* sequential test) by presenting the source test either in immediate sequence to item recognition (as standard in source-monitoring research) or the source test followed as a separate block after full completion of the item recognition test to separate these processes in time (as our baseline). The blocked format served to provide relatively pure measures of item and source memory, respectively: Even if participants predicted that they will be tested for source at some point, they must not have prepared for it as much during the item test, because the source would only become relevant much later.

We derived separate predictions depending on whether there is a temporal sequence or a (partial) temporal overlap between item and source memory. Intuitively, one would herein expect that the source test would generally create more curvature than its item test because source memory needs more detailed recollection, while recency or non-specified familiarity is sufficient to decide item recognition (Johnson et al., 1997; Yonelinas, 1999). This should particularly show in the blocked format, which more purely measures item versus source retrieval courses, as reasoned above. However, as the direct mapping of mouse trajectories on source monitoring has not yet been explored, we cannot be sure whether this assumed greater required differentiation of source memory (Johnson et al., 1993) indeed translates to more curvature in mouse movements. More crucial to our research question is the comparison of item and source trajectories, regardless of whether they show differential curvatures, between the standard and blocked format:

Hypothesis (H)1. If we observe no significant interaction between memory type and test format, that suggests a strictly serial temporal sequence between item and source memory. That means the difference (or non-difference) between the item and source trajectory curvature is the same and does not matter if tested in succession or in a blocked manner.

H2. In case of a significant interaction, we indicated looking at the patterns of the standard format more closely. If in this format the difference between the source and item trajectory curvature is less pronounced (or even null or in the reverse direction) than in the blocked format, that would speak for a (partial) temporal overlap of item and source memory. Put differently, this would suggest that during the item test of the standard format, participants already began retrieving the source in addition to the item, since they knew they would be tested for source memory following their “old” answer. Consequently, part or all of the curved trajectory shown in the blocked source test was outsourced to the item test in the standard format.

## Method

The present study was preregistered in the Open Science Framework (OSF). All materials, including experiment scripts, and results (also supplementary analyses), are available online at https://osf.io/jkrx6/.

### Participants

Power analysis using the G*Power-3 software (Faul et al., 2007) for an ANOVA analysis of the aggregate MAD values indicated that a sample size of 60 (i.e., 30 per test format condition) would provide .80 power to detect a medium-sized (i.e., *f* = .25) within-subjects effect (i.e., of memory type: item vs. source) as well as a medium-sized (i.e., *f* = .25) interaction between memory type and test format even when conservatively assuming only a .10 correlation between the repeated measures. As these effects were of most interest to our research question, we thus collected data until *n* = 30 was reached for each source-monitoring test format. We acknowledge that our design was only sufficiently powered to detect a large (i.e., *f* = .40) between-subjects effect (i.e., of test format: blocked vs. standard sequential).

Sixty-three German-speaking subjects participated in the experiment. Three participants were excluded from the data analysis because they did not comply with the requirements of the experiment and did not follow the instructions, or else due to technical problems. Analyses were carried out with the remaining 60^1^ (43 female, 17 male; *M*_*age*_= 24.92 years, age range = 18–30 years). They were either native Germans (38 participants) or learned German before the age of 6 years (22 participants). The majority (53 participants) indicated a preference for the right hand and all 60 participants reported using a computer mouse with the right hand.

Younger adults were recruited either via the electronic SONA system of the University of Mannheim or via social media groups. We posted our exclusion criteria (i.e., German native or learned German before the age of 6 years; age 18–30 years; no diagnosed/on-going mental health/illness condition) while advertising the study and participants anonymously reported on them in the study. Ten participants were tested in our lab. However, due to the COVID-19 pandemic, we tested the remaining majority of participants remotely if they were willing to install the required software and plug in on their personal computer/laptop under our instructions via video chat. The experimental task lasted approximately 45 min. Participants received either course credit or payment according to our department-set rate of 8€/h. If remote testing took much longer for unforeseen technical issues during installation, we naturally compensated them for the full time.

### Design

The design was a 2 (test format: the blocked sequential test format, the standard sequential test format) × 2 (memory type: item memory, source memory) mixed factorial with memory type as a within-subjects factor and test format as a between-subjects factor.

It is also crucial to note here that spatial position of study words (top vs. bottom) was manipulated within-subjects. Half of these words were presented centered on the top of the computer screen, the other half centered on the bottom. However, as this was preregistered, we did not expect differences in word or position memory between these screen positions and, after ensuring this held in the current data (see Online Supplementary Material), collapsed across this factor in data analysis.

### Materials

The item set consisted of 108 emotionally neutral German nouns that were randomly chosen from the Berlin Affective Word List (BAWL-R; Võ et al., 2009) after controlling for certain characteristics (valence: -1.5 to 1.5, arousal: < 3, imageability: > 2, word length: 4–8, number of syllables: 2–3, and frequency: 20–150). From this set, words were randomly assigned to serve as study items (on the top or on the bottom) or distractors for each participant.

### Procedure

Automatic stimulus display and data collection were controlled with *OpenSesame* software (Mathôt et al., 2012; version used: legacy backend 3.2.8), using the *mousetrap* plug-in (Kieslich & Henninger, 2017). The experiment was conducted full-screen at a resolution of 1,920 × 1,080 pixels running Windows 10. Remote data collection was limited to individuals whose computer/laptop had the same system qualities and a physical computer mouse (i.e., not touchpad). Thus, these technical features did not differ between the lab and remote testing. The mouse sensitivity settings were left at the system defaults (medium speed, with acceleration enabled). For remote testing, we checked these settings by interacting directly with participants via video chat. Mouse cursor movements were recorded every 10 ms.

Participants were randomly assigned to the experimental conditions upon arrival at the laboratory or recruitment for remote testing. We ensured a comparable distribution across the between-subject groups (i.e., test formats) for lab testing versus remote testing. Before the experiment, participants were requested to complete an informed consent form within the experiment program.

The main experimental task consisted of three phases including a study phase, filler task, and test phase. All stimuli and instructions were printed with 36-point Arial font in black against a white background throughout the experiment. Critically, to increase memory-based test responses, item and source learning were intentional, that is participants were explicitly told before the study phase that they should learn both words and their screen positions, and that they would be informed later which exactly they will be tested on (see below for further details on the instructions). In the actual study phase, 72 German nouns (first letter capitalized in accordance with German spelling) appeared in the upper or lower part of the screen (50% on the top vs. the bottom of screen) for 4 s. A centered fixation dot appeared for 250 ms and a blank screen lasting for 250 ms preceded each stimulus (i.e., 500-ms inter-stimulus interval, in total). Selection of study words, their assignment to the screen positions, and the presentation order were randomized anew for each participant. Participants saw two (fixed) additional primacy buffer items in the study phase that were presented first, one on the top and one on the bottom, and that then along one more (fixed) distractor word served in the practice test.

**Fig. 1:**
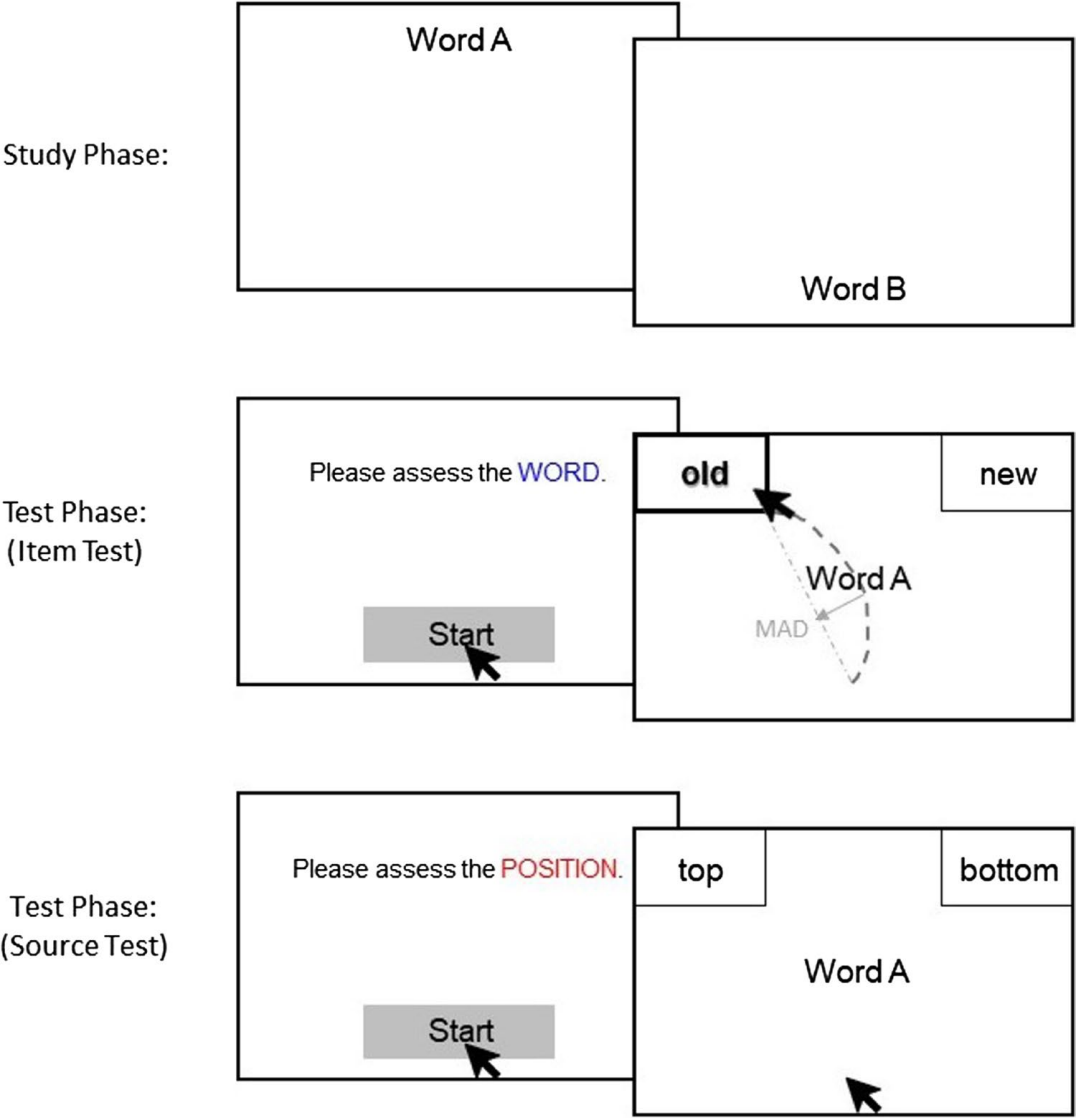
Mouse-tracking procedure for the source-monitoring paradigm. *Note.* In the study phase, participants saw a number of words (i.e., items) presented either at the top or at the bottom of the screen (i.e., sources). In the test phase, they decided on old/new recognition and source attribution sequentially after a start screen. While participants in the standard format decided item and source decision consecutively for each item upon old response, participants in the blocked format were first asked about their item decision for all items, and then they were asked to indicate the source of the recognized stimuli

After the study phase, in order to eliminate the recency effect, participants worked on a 3-min filler task that consisted of basic mathematical equations. Following the filler task, participants were presented with the source-monitoring test, formatted according to their condition. Although they were instructed to respond as quickly and as accurately as possible, all test responses were self-paced. We deemed it crucial that there was no time pressure so that memory processes had ample time to unfold and influence response movements. Before the test session, participants in the standard sequential test condition (cf. Dodson & Johnson, 1993; Marsh et al., 2006; Marsh & Hicks, 1998) were informed that they would be tested for their item memory first, immediately followed by a test for their source memory if they indicated that a word was old in the first step. The 72 old (i.e., 36 top and 36 bottom) and 36 new words were presented in a different random order for each participant. Each test trial began with a start button in the bottom center of the screen (see Hehman et al., 2015; Kieslich & Henninger, 2017). Immediately after clicking on this start button with the computer mouse, a word was shown in the screen center, and the mouse cursor was reset to the exact center of the start button at the bottom center, which enabled us to align each response with an equal starting point. Participants indicated their response as *old* or *new* by clicking on one of the two buttons located in the top-left and top-right corners of the screen (assignment of response options to button location counterbalanced across participants). In this condition, if participants indicated that a word was old, they were next asked to indicate whether it was shown at the top or the bottom of the screen. Similarly, they started this trial of the test by clicking on the start button, and the same word that they just classified as old appeared again in the screen center, with the mouse centered on the start button on the bottom. They indicated their response as either *top* or *bottom* by clicking on one of the two buttons located in the top-left and top-right corners of the screen (assignment again counterbalanced across participants). However, if they responded with *new* in the first item query, the next test trial began immediately. Thus, after they clicked on the start button, a different word appeared in the screen center, and they were again asked to decide whether it was old or new. In the blocked sequential test condition (cf. Fox & Osth, 2022; Osth et al., 2018; Starns et al., 2013), however, before the test session, participants were informed that only their item memory would be tested at this point, and that position is irrelevant for the responses. No mention of the later source test was made to minimize source retrieval at this stage. Thus, in this condition, participants were firstly questioned about whether the words were old or new. The test set-up was exactly the same as in the standard test condition just described, but with the crucial difference that independent of whether *old* or *new* was the given response, no source question was posed (i.e., it immediately proceeded with the next test word as for new responses in the standard test condition). Once participants in the blocked test condition had completed the item test for all words, they were then presented again with all words they previously judged as *old* in the order they had responded and this time asked to indicate their sources, with the same mouse-tracking procedure as in the source test of the standard test. The experimental procedure is illustrated in Fig. 1.

In all tests, participants had to indicate their response by clicking on one of the two buttons located in the top-left and top-right corners of the screen to proceed from each trial. Thus, they needed to answer each trial to complete the experiment, preventing any missing data. Assignment of the response options (old vs. new; top vs. bottom) to the buttons in the top-left versus top-right corner of the screen was counterbalanced across participants. Because counterbalancing was done between participants, the labeling of the response buttons stayed fixed across trials throughout an experiment session to avoid confusion. Participants were additionally informed before the test phase about which option would be presented on which side. Accuracy scores and mouse movements were automatically recorded via the *OpenSesame* scripts. At the end of the experiment, participants indicated their demographic information (i.e., age and gender) and indicated their proficiency in German, their handedness and, more specifically, the hand they use for moving the mouse (cf. Kieslich et al., 2020).

## Results

We fully followed our pre-registered plan for data preparation and analysis. After reporting the mouse-tracking analyses based on aggregated trajectory curvatures, as planned in our pre-registration, we additionally report more fine-grained analyses based on individual trajectories (cf. D. Wulff et al., 2019; Kieslich et al., 2020). We performed all mouse-tracking analyses in *R* (R Core Team, 2018)^2^.

We filtered the mouse-tracking data to analyze only correctly answered trials. Thus, correct source attributions upon correct target detections (41% of targets across both conditions) were included. The total number of accurate trials entering the following aggregated analyses is 933 for the blocked format (*M* = 31 trials per participant, range = 13–54) and 827 for the standard format (*M* = 28 trials per participant, range = 11–50). Information in the Online Supplementary Material additionally shows the multinomial processing tree (MPT) model of source monitoring (Bayen et al., 1996) for the present data as a more fine-grained analysis of the memory processes involved.

### Analyses based on aggregated trajectory curvatures

Trajectory measures were derived as follows using the *mousetrap* R package (Kieslich et al., 2016). From the raw data, we extracted the x-y coordinates of the cursor across the interval from the start of the test screen until the response in 10-ms steps (Kieslich et al., 2019). As the correct answer was sometimes to the left and sometimes to the right, we remapped all trajectories to one side. Thus, we flipped all trajectories that ended on the right response option to the left. Of course, given the variation in (self-paced) response times, the total number of recorded coordinates varied across trials. Therefore, we applied the time-normalization function, which divides each trajectory into 101 equally spaced time steps. Then, we computed the MAD for each trajectory (Kieslich & Henninger, 2017).

**Fig. 2:**
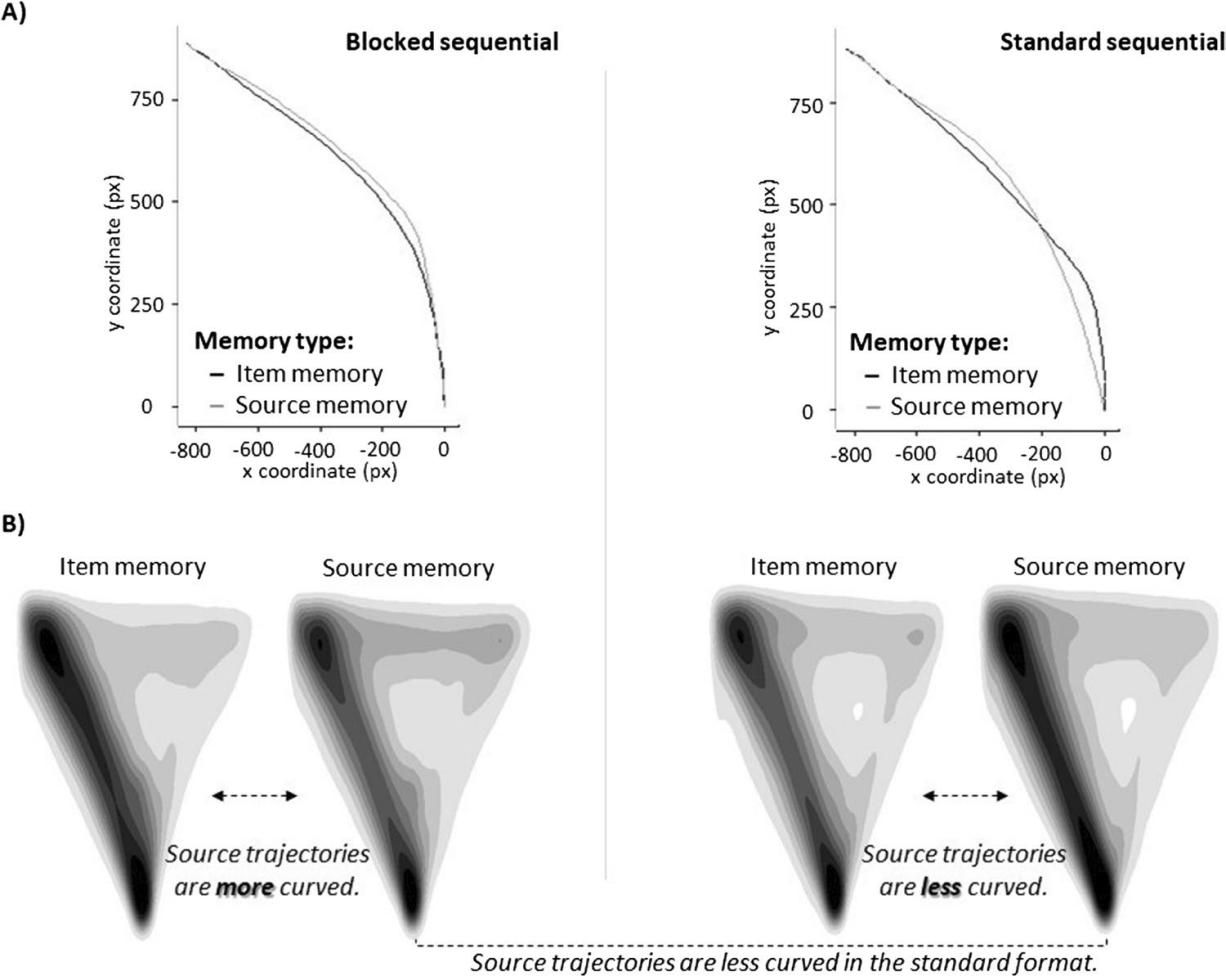
Aggregate and individual mouse trajectories. *Note.* Left and right panel indicate the mouse trajectories in the blocked and standard test format, respectively. (**A**) Aggregated trajectory curvatures. All answers were flipped to the left and time-normalized. (**B**) Smoothed heat maps of the individual trajectories underlying the aggregate curvatures. This is a graphical illustration for analyzing the trajectories at the trial-level. Darker colors indicate higher density (see also Kieslich et al., 2020). Although the straight trajectories are less common (i.e., trajectories are more curved) in the source test of the blocked format compared to its item test, the reversed pattern is displayed for the standard format in which its source test includes more straight trajectories (i.e., trajectories are less curved) relative to its item test

After preprocessing data, we aggregated the trajectories per memory type, first within and then across participants, and separately for test formats. Figure 2A displays the aggregate trajectories that appear to only differ in details. To test for differences statistically, we conducted a repeated-measures ANOVA using the aggregated MAD values per participant with the within-subjects factor memory type and the between-subjects factor test format. Neither the main effects of memory type, *F* < 1, nor test format, *F*(1, 58) = 1.06, *p* = .307, ${\upeta}_{\textrm{p}}^2$= .02, nor their interaction, *F*(1, 58) = 2.76, *p* = .102, ${\upeta}_{\textrm{p}}^2$= .05, were significant. However, there was some variation around the mean estimates as well as a descriptive trend capturing that either item or source trajectories were numerically more curved differed by test format (Table 1). We additionally performed a Bayesian repeated-measures ANOVA with JASP (Wagenmakers et al., 2018) and assessed the likelihood of data under one alternative hypothesis relative to the null hypothesis on the basis of Bayes factors (*BF*_10_). We report the Bayes Inclusion Factor (*BF*_Incl_) across matched models. There was weak-to-moderate evidence for the null hypothesis for the main effects of test format (*BF*_Incl_ = 0.36) and memory type (*BF*_Incl_ = 0.25), but the results suggested ambiguous evidence regarding the interaction (*BF*_Incl_ = 1.01), warranting further analyses based on the trial-level to test whether our aggregate MAD results were an artifact of condensing the individual trajectories.

**Table 1:** Means (and standard deviations) for aggregated MADs (maximum absolute deviation toward the non-chosen option), and paired t test for the comparison of memory type

Condition	*N*	Item memory	Source memory	*t* test		
				*t*	*p*	*d*
Blocked sequential	30	289.08 (172.26)	316.30 (162.49)	0.68	.505	0.12
Standard sequential	30	299.02 (195.62)	234.04 (160.30)	-1.70	.099	-0.31

*Note:* MAD values were aggregated per participant and memory type in each test format condition. More curved (less straight) trajectories are represented by increased MAD values

### Analyses based on individual trajectories

For MAD values, a linear mixed model accounts for intraindividual variation in a more efficient way than the current averaging per person does (cf. L. Wulff & Scharf, 2020). We conducted our linear mixed model analyses^3^ with the *lme4* (Bates et al., 2015) and the *lmerTest* R package (Kuznetsova et al., 2017). We included memory type and test format as effect-coded predictors, their interaction as well as a random intercept^4^ per participant (Table 2). Critically, the results showed a significant interaction of both predictors, *b* = 120.87, *t*(3456.88) = 4.47, *p* < .001. Next, we compared the model with and without the interaction to verify whether the interaction is needed to explain the data (e.g., Baayen et al., 2008). The likelihood ratio test showed that the model including the interaction explained significantly more variance, χ^2^ (1) = 19.89, *p* < .001.

**Table 2:** Linear mixed model with trial-based MADs (maximum absolute deviation toward the non-chosen option) as the dependent variable

Predictors	*b*	*SE*	*Df*	*t*	*p*
Intercept	281.80	17.29	56.35	16.30	< .001
Memory type	-2.59	13.54	3,456.88	-0.19	.849
Test format	40.97	34.57	56.35	1.19	.241
Memory type × test format	120.87	27.07	3,456.88	4.47	< .001

*Note:* We included the effect-coded predictors memory type (item memory = -.5, source memory = .5) and test format (blocked sequential test format = .5, standard sequential test format = -.5) as well as their interaction. Participants were included as random intercepts. *b* = beta-weight of effect, *SE* = standard error, *df* = degrees of freedom, *t* = *t* values, *p* = *p* values

To follow up on this interaction, we conducted post hoc pairwise comparisons (*p* values were corrected with the Bonferroni-Holm procedure) using the *emmeans* package (Lenth, 2019). In the standard format, there was a significant difference between the item and source trajectories such that trajectories were less curved in the source test, *t*(3456.9) = 3.20, *p* = .008. In the blocked format, however, this difference was significant in the direction of more curved trajectories in the source test, *t*(3456.9) = -3.12, *p* = .009 (Fig. 2B). While the source trajectories were significantly less curved in the standard format than the blocked format, *t*(74.9) = 2.73, *p* = .031, the item trajectories did not differ significantly across the test formats, *t*(74.9) = -0.52, *p* = .733. Overall, these results demonstrate that in the standard format, trials in the source test led to less curved trajectories relative to its item test, whereas the corresponding difference was in the opposite direction in the blocked format, and that this significant interaction across the conditions seems to be mainly driven by the source trajectories.

## Discussion

For comparison purposes, we employed a blocked test format not typically used in source monitoring research (but see Fox & Osth, 2022) to gain insight into item and source memory processes in the commonly used standard source-monitoring test format. Although the aggregated mouse trajectories indicated no significant difference across tests, the trial-level analyses revealed that trajectories were more curved in the source than in the item test of the blocked format. In the standard format, this difference was reversed, with source showing less curved trajectories than item.

The observed differences confirm the theoretical expectation that the more difficult, recollection-based source memory (with its higher level of differentiation; Johnson et al., 1993) is associated with more curvature than the less difficult, familiarity-based item memory, but only if the source test was delayed from the item test. On the basis of our preregistered hypotheses, this suggests that people may be able to retrieve source information parallel to item information in preparation of the source test in the standard test format. However, we critically discuss this finding and outline open questions as follows. Probing the interaction between memory type and test format further showed that the source trajectories were less curved if tested in immediate sequence to item recognition than tested as a separate block, whereas the item trajectories did not significantly differ by test format. That hinders us from going further merely on the parallelity account and raises another possible explanation of item familiarity serving as a basis for source decision.^5^ Specifically, the consecutive testing in the standard format may result in *easier* source retrieval when participants are already in the state of item recognition. Put differently, source processing may not commence during the item test of the standard format (as portrayed by the parallelity account) but rather start with the source query. However, being already in the state of item recognition may just facilitate reaching the state of source attribution. Vice versa, while working on the source test of the blocked format, participants likely did not suppress item information completely, and recognized the item again. This may potentially explain why only the source trajectories differed across the test formats without any costs to the item trajectories. Albeit desirable for further disentanglement in future studies, both of these possibilities suggest close links of item and source retrieval courses, leaving open the challenge of the current research focus. Overall, the time-course question invites a closer investigation of possible patterns of parallelity together with the debate surrounding the serial sequence of item and source memory (e.g., Malejka & Bröder, 2016; Osth et al., 2018).

Mouse-tracking brings a new perspective to this time-course question and provides a useful analytic technique to look at how item and source decisions evolve over time, which is the genuine dynamic process described theoretically by Johnson et al. (1993) under the concept of differentiation. Here, we focused on how straightforwardly participants develop their response in the source-monitoring paradigm as measured by one of the curvature metrics, namely, MAD.^6^ Due to their previously demonstrated link to source memory (L. Wulff & Scharf, 2020), we analyzed the MAD values but with a careful consideration of their interpretation. There are varied terms used in the literature describing what trajectories reveal, such as conflict/activation between competing options or one’s tentative commitment/attraction to a certain response (Schoemann et al., 2021). For the special case in which L. Wulff and Scharf (2020) investigated stereotype consistency (i.e., consistent vs. inconsistent sources) on source monitoring, the activation of the non-chosen response option can be an indicator of “cognitive conflict.” However, in the current study, there is no systematic schema to guide guessing (Bayen et al., 2000) as our aim was to investigate memory processes by simply manipulating the position information, which is regarded as a relatively superficial source cue. Hence, even though the MAD reflects uncertainty in the source monitoring process (L. Wulff & Scharf, 2020), it is as yet unclear whether that is an index of conflict or confidence (cf. Papesh & Goldinger, 2012). Which aspects of mouse trajectories map onto which particular processes depends on the given task (Freeman et al., 2011). As our study seems to be only the second application of mouse tracking to source monitoring, certainly more research is needed.

The present study could guide further research regarding the qualitative nature of memory processing in source monitoring. The results do clearly show that there are pronounced interindividual differences in item and source memory mouse trajectories. Thus, further research should carefully focus on the examination of individual trajectories rather than aggregated trajectory curvatures, as has also been suggested for mouse-tracking analyses in other cognitive paradigms (Kieslich et al., 2019).

## Conclusion

Mouse tracking is an insightful way to examine memory processes in source monitoring by exploring the temporal *development* of memory processes over time. Although the evidence is not fully conclusive on the partially overlapping parallel processes of item and source memory, the observed trajectories suggest that querying for item and source memory in immediate succession on a standard source-monitoring task smooths source retrieval compared to when the source is queried in a separate test block. Yet, to draw definite conclusions regarding the possibility of parallel item and source retrieval – especially with regard to the degree of parallel overlap possible – further evidence based on complementary routes from various methodological and analytic techniques is needed.

### Supplementary Information

ESM 1 (DOCX 65 kb)

## Data Availability

The datasets analyzed during the current study are available in the OSF repository and can be accessed via the at https://osf.io/jkrx6/.

## Footnotes

1 No participant had fewer hits than false alarms.

2 Analyses and visualization of the mouse-tracking data relied on the ggplot2 package (Wickham, 2016), the dplyr package (Wickham et al., 2019), the tidyr package (Wickham & Henry, 2019), the afex package (Singmann et al., 2018), and the MBESS package (Kelley, 2017).

3 Although we preregistered that we would explore the individual trajectories, we did not specify this linear mixed model analysis. We thank an anonymous reviewer for suggesting this analysis. It is thus worth noting that the sample size planning was based on our planned analyses at the aggregated -level only.

4 We also tried a linear mixed model including words as an additional random intercept, but this model was overfitted resulting in a singularity warning (see our R code in the OSF). Thus, we simplified the random structure by removing the intercept of the word (e.g., Gatti et al., 2022). Note that we carefully selected our words as an initial step to control for the noise of items (see Materials section). Further, via the OpenSesame scripts, assignment of the words as targets and lures as well as assignment of targets to the sources were randomized anew across participants, making each participant tied to their own unique random set of items.

5 We thank an anonymous reviewer for bringing up suggesting this alternative interpretation.

6 As preregistered, we conducted our analyses on the MAD values. However, interested readers can still find the dataset including the other mouse-tracking metrics as well as response times (RTs) per trial in the OSF.
