# Supplementary material for: The temporal development of memory processes in source monitoring: An investigation with mouse tracking
Source: Psychon Bull Rev. 2023 May 3;30(6):2305–14. doi: 10.3758/s13423-023-02289-z (PMC10156421; doi:10.3758/s13423-023-02289-z)
Supplement: Supplementary file 1 — (DOCX 65 kb) [file 13423_2023_2289_MOESM1_ESM.docx]

# **Supplementary Information**

**MPT Model-Based Analyses**

The two-high-threshold multinomial processing tree model of source monitoring (2HTSM; Bayen et al., 1996) is advised for obtaining guessing-corrected measures of memory for item and source and separate estimations of guessing bias (for extensive discussion, see also Bröder & Meiser, 2007; Erdfelder et al., 2009). Thus, in the current study, we employed the 2HTSM for disentangling cognitive processes on the basis of response frequencies. Multinomial model fit was assessed via maximum likelihood estimation methods and the *G*^2^ statistic. A *p* value above .05 indicates that the model explains the data well. Parameter differences were evaluated based on the chi-square difference test statistic Δ*G*^2^ for model comparison. If the *p* value for the difference test statistic is below .05, the parameter difference is considered significant. Note that we preregistered our MPT model-based analyses but did not indicate any specific hypotheses as the comparisons of memory parameters across the test formats were not the main focus of interest in this mouse-tracking study, so here we rather investigated them as exploratory.

For the current study, the most relevant parameters of the 2HTSM are *D* and *d*, standing for item and source memory, respectively. We performed joint MPT model-based analyses to separately measure source-monitoring processes in each condition but in one overarching joint model allowing for comparison. We used the *multiTree* software (Moshagen, 2010) for model fitting and parameter estimation. We followed the identifiable submodels of the 2HTSM (see Bayen et al., 1996, for a detailed overview of alternative model versions). We implemented the most basic and restrictive Submodel 4, as illustrated in Figure S1, with four free parameters (i.e., *D* [assuming equal detection of items presented on the top or bottom and new distractor items], *d* [assuming equal probability of remembering the top or bottom source], *b*, *g* [assuming equal source guessing when source memory fails independent of item recognition status]) which fit the data, *G*^2^(4) = 4.46, *p* = .347. We estimated item and source memory (parameters *D* and *d*; item and source guessing, parameters *b* and *g*, were also estimated but were not of central interest here) across the test formats (see Table S1). After defining our baseline model, we tested the effect of test format by implementing equality restrictions between parameters.

First, we tested the effect of test format on item memory by restricting parameter *D* to be equal across conditions. These restrictions significantly decreased model fit, Δ*G*^2^(1) = 12.37, *p* < .001. Then, we tested the effect of test format on source memory by restricting parameters *d* to be equal across conditions. Again, the model fit became significantly worse, Δ*G*^2^(1) = 7.20, *p* = .007. Overall results suggest that item memory was better in the blocked format while source memory was better in the standard format. In line with Mulligan et al. (2010), item memory was better in the blocked format because the instructions of source monitoring might have changed the sensitivity of old/new recognition in the standard format. Likewise, it is not surprising that source memory was better in the standard format because participants knew they would be tested next for sources upon their “old” answer, and they might have used more stringent criteria at first (cf. Dodson & Johnson, 1993). Next, we tested the effect of test format on item guessing (parameter *b*), and the model fit became significantly worse, Δ*G*^2^(1) = 29.31, *p* < .001. We found a pronounced stronger bias to guess old in the blocked format, which would be expected since participants in the standard format knew they would be tested on source for each “old” response, and thus they only said “old” if they were quite sure. However, participants in the blocked format were not informed about the upcoming source test; consequently, they were more liberal to guess old upon no item detection. Finally, we tested the effect of test format on source guessing (parameter *g*), and we found no significant difference, Δ*G*^2^(1) = 0.32, *p* = .574. However, source guessing averaged across the test formats, *g* = .46, 95% CI [.44, .48], was slightly but significantly below .5, Δ*G*^2^(1) = 11.41, *p* = .001. That is, there was a bias to guess bottom rather than top, but more importantly, this guessing tendency was comparable across the test formats.

## **Supplementary References**

Bayen, U. J., Murnane, K., & Erdfelder, E. (1996). Source discrimination, item detection, and multinomial models of source monitoring. *Journal of Experimental Psychology: Learning, Memory, and Cognition*, *22*(1), 197-215. <https://doi.org/10.1037/0278-7393.22.1.197>

Bröder, A., & Meiser, T. (2007). Measuring source memory. *Zeitschrift für Psychologie / Journal of Psychology*, *215*(1), 52-60. <https://doi.org/10.1027/0044-3409.215.1.52>

Dodson, C. S., & Johnson, M. K. (1993). Rate of false source attributions depends on how questions are asked. *American Journal of Psychology*, *106*(4), 541-551. <https://doi.org/10.2307/1422968>

Erdfelder, E., Auer, T.-S., Hilbig, B. E., Aßfalg, A., Moshagen, M., & Nadarevic, L. (2009). Multinomial processing tree models. *Journal of Psychology*, *217*(3), 108–124. <https://doi.org/10.1027/0044-3409.217.3.108>

Moshagen, M. (2010). multiTree: A computer program for the analysis of multinomial processing tree models. *Behavior Research Methods*, *42*(1), 42-54. <https://doi.org/10.3758/BRM.42.1.42>

Mulligan, N. W., Besken, M., & Peterson, D. (2010). Remember-know and source memory instructions can qualitatively change old-new recognition accuracy: The modality-match effect in recognition memory. *Journal of Experimental Psychology, 36*(2), 558-566. <https://doi.org/10.1037/a0018408>

## **Table S1**

*Parameter Estimates and Confidence Intervals for the Four-Parameter Two-High-Threshold MPT Model of Source Monitoring Under Different Conditions of Test Format*

|  | Model parameters | | | |
| --- | --- | --- | --- | --- |
| Test format | *D* | *b* | *d* | *g* |
| Blocked sequential | .44 [.40, .47] | .42 [.39, .45] | .44 [.36, .52] | .47 [.44, .50] |
| Standard sequential | .36 [.32, .39] | .30 [.28, .33] | .61 [.51, .70] | .45 [.42, .49] |

*Note.* The presented model parameters are probability estimates that can range from 0 to 1. *D* = item memory; *b* = item guessing (chance level is .5); *d* = source memory; *g* = source guessing (estimates higher than the chance level of .5 indicate guessing bias towards “top”; estimates lower than .5 indicate guessing bias towards “bottom”). Brackets indicate 95% confidence intervals.

## **Figure S1**

*Two-High-Threshold MPT Model of Source Monitoring Adapted to Our Source Manipulation*

**
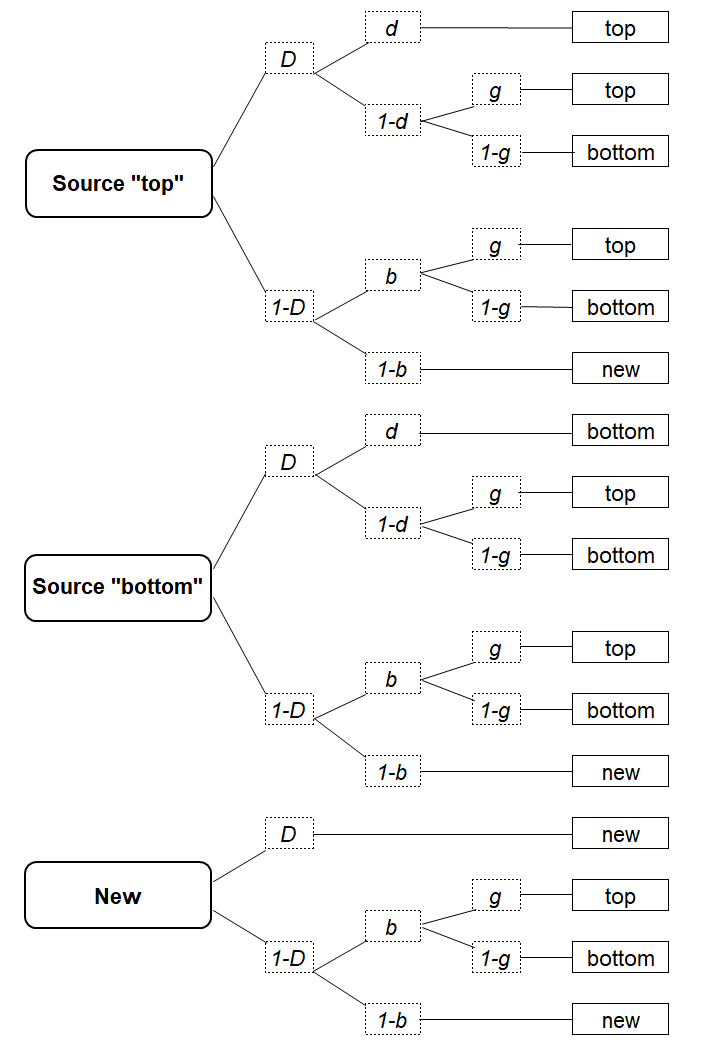
**

*Note.* Labels in the leftmost refer to items presented on the source-monitoring test. Labels in the rightmost refer to observed responses. Labels within the branches surrounded by dashes refer to latent cognitive states. *D* = probability of detecting a specific item as old (or a distractor as new); *d* = probability of correctly remembering the source of that item; *g* = probability of guessing that an item was presented by the source “top”; *b* = probability of guessing that an item is old. Adapted from Bayen et al. (1996, Model 4).
